# *“Tell us what’s going on”*: Exploring the information needs of pregnant and post-partum women in Australia during the pandemic with ‘Tweets’, ‘Threads’, and women’s views

**DOI:** 10.1371/journal.pone.0279990

**Published:** 2023-01-13

**Authors:** Cassandra Caddy, Marc Cheong, Megan S. C. Lim, Robert Power, Joshua P. Vogel, Zoe Bradfield, Benjamin Coghlan, Caroline S. E. Homer, Alyce N. Wilson

**Affiliations:** 1 Burnet Institute, Melbourne, VIC, Australia; 2 School of Computing and Information Systems, University of Melbourne, Parkville, VIC, Australia; 3 School of Population and Global Health, University of Melbourne, Parkville, VIC, Australia; 4 Curtin University, Bentley, WA, Australia; 5 King Edward Memorial Hospital, Subiaco, WA, Australia; The Islamia University of Bahawalpur Pakistan, PAKISTAN

## Abstract

**Introduction:**

The provision of maternity services in Australia has been significantly disrupted in response to the COVID-19 pandemic. Many changes were initiated quickly, often with rapid dissemination of information to women. The aim of this study was to better understand what information and messages were circulating regarding COVID-19 and pregnancy in Australia and potential information gaps.

**Methods:**

This study adopted a qualitative approach using social media and interviews. A data analytics tool (TIGER-C19) was used to extract data from social media platforms Reddit and Twitter from June to July 2021 (in the middle of the third COVID-19 wave in Australia). A total of 21 individual semi-structured interviews were conducted with those who were, or had been, pregnant in Australia since March 2020. Social media data were analysis via inductive content analysis and interview data were thematically analysed.

**Results:**

Social media provided a critical platform for sharing and seeking information, as well as highlighting attitudes of the community towards COVID-19 vaccines in pregnancy. Women interviewed described wanting further information on the risks COVID-19 posed to themselves and their babies, and greater familiarity with the health service during pregnancy, in which they would labour and give birth. Health providers were a trusted source of information. Communication strategies that allowed participants to engage in real-time interactive discussions were preferred. A real or perceived lack of information led participants to turn to informal sources, increasing the potential for exposure to misinformation.

**Conclusion:**

It is vital that health services communicate effectively with pregnant women, early and often throughout public health crises, such as the COVID-19 pandemic. This was particularly important during periods of increased restrictions on accessing hospital services. Information and communication strategies need to be clear, consistent, timely and accessible to reduce reliance on informal and potentially inaccurate sources.

## Introduction

Australia recorded its first case of COVID-19 in late January 2020 with community transmission occurring soon after [1]. In response, the Australian government rapidly implemented several risk reduction measures including travel restrictions, wearing of masks, imposing density limits in public and private spaces and periodic community lockdown measures [1]. Many non-essential health services were closed or where possible, delivered online. Despite being an essential service, maternity and newborn care was significantly disrupted by these changes. Face-to-face antenatal care was replaced by telehealth in many jurisdictions, antenatal education classes were cancelled or moved online, and limits on the number of support people who could attend ultrasound scans, labour, birth and the postnatal ward were introduced [2–4].

Whilst pregnant women are not more likely to contract COVID-19 compared to non-pregnant women, if they do so they are at higher risk of death or developing severe disease from SARS-CoV-2 infection, including higher rates of intensive care unit (ICU) admission and invasive ventilation when compared to non-pregnant women [5]. Globally, increasing maternal mortality rates have been seen in location such as Brazil and Mexico as a result of the pandemic [6, 7]. In addition, babies of women with COVID-19 infection during pregnancy have higher rates of preterm birth and admission to neonatal ICU [5, 8]. As SARS-CoV-2 spread rapidly worldwide, health systems were in a constant state of flux–reacting, adapting and mitigating in response to the novel coronavirus [9]. Clear, timely and targeted information was essential to support pregnant women in making healthcare-related decisions during an uncertain and rapidly evolving time. Social media represented an ideal platform to spread information quickly and has been used to rapidly disseminate information during previous public health emergencies and disease outbreaks, including Ebola, H1N1 (swine flu) and MERS-CoV outbreaks [10–13]. In addition, social media analytics have been used to better understand public perceptions and responses to emerging infectious diseases [14].

Whilst pregnant women using social media to access information about pregnancy, birth and caring for their baby is not a new phenomenon [15–18] the novel nature of the pathogen in the setting of a global pandemic created the perfect storm for an ‘infodemic’ [17]. This infodemic led to an abundance of false and misleading information on digital platforms, creating barriers to women’s access of accurate information [17]. Several studies have documented shortcomings in health communication related to pregnancy and childbirth during the pandemic, leaving many women and families feeling anxious and isolated [3, 4, 19–22].

Australia was one of the few countries globally that was able to bring community transmission of COVID-19 to zero in 2020 [23] and its second largest city, Melbourne, was renowned for being the most ‘locked down’ city in the world [24]. As such, Australia is a unique setting in which to explore the use of social media as a source of information for pregnant and postnatal women during the pandemic. This study aimed to describe what information about COVID-19 and pregnancy was being shared on two social media platforms in Australia, Twitter and Reddit, and identify critical information gaps for pregnant women during the COVID-19 pandemic, to inform future communication strategies during public health emergencies.

## Methods

### Study design

An exploratory qualitative study design was used, comprising social media analysis and individual in-depth interviews. This approach allowed researchers to explore community attitudes to COVID-19 during and after pregnancy as expressed on social media, as well as gaining an in-depth understanding of the attitudes and perceptions of those with lived experience of being pregnant during the pandemic. Phases occurred concurrently from June to July 2021. Prior to June 2021, levels of COVID-19 community transmission in Australia had been relatively low compared to international settings, however shortly after Australia’s two most populous cities, Melbourne and Sydney, both experienced significant surges of community transmission and spent extended periods in lockdown. Alfred Hospital Ethics Committee provided ethics approval (ID:172/21).

### Social media

#### Sample and recruitment

**Fig 1:**
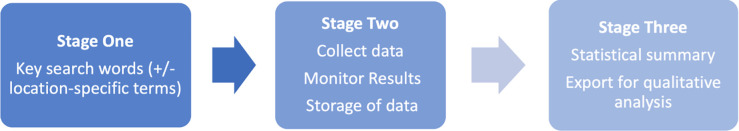
TIGER-C19 data capture methodology.

The data analytics tool TIGER-C19 (Timely Integration of user-GEnerated Responses to C19) (Fig 1) was used to extract publicly available posts containing selected key terms from two social media platforms–Reddit and Twitter. Both platforms allow users to create posts and discussion in a publicly available forum. Reddit consists of multiple online communities or ‘subreddits’ specific to a topic of interest [25]. Two popular methods of sharing or liking posts on these platforms include retweeting (Twitter) and upvoting (Reddit). Retweeting involves the sharing of a Twitter post by another user, upvoting (Reddit) allows users to ‘like’ others posts/threads and can indicate the level of approval of the post from other users. Use of Reddit in Australia is increasing, and Australian users comprise of the platforms fourth largest user base [26]. Extracted posts from Reddit came from two Australian subreddits related to COVID-19 and pregnancy. Posts from Twitter and Reddit were eligible for data collection from June to July 2021 if they met the inclusion criteria: written in English and relevant to the research phenomenon. Posts were excluded if they contained no expression of personal opinion or comment (for example re-tweeting a news article with no added text), the meaning of the post was indiscernible or if they were not related to the Australian context. This was filtered by both the TIGER-C19 data analytics tool and the researcher when reviewing collected posts. Details of TIGER-C19 methodologies have been documented previously [27]. Data collection complied with the terms and conditions of social media platforms Reddit and Twitter.

#### Data collection

**Fig 2:**
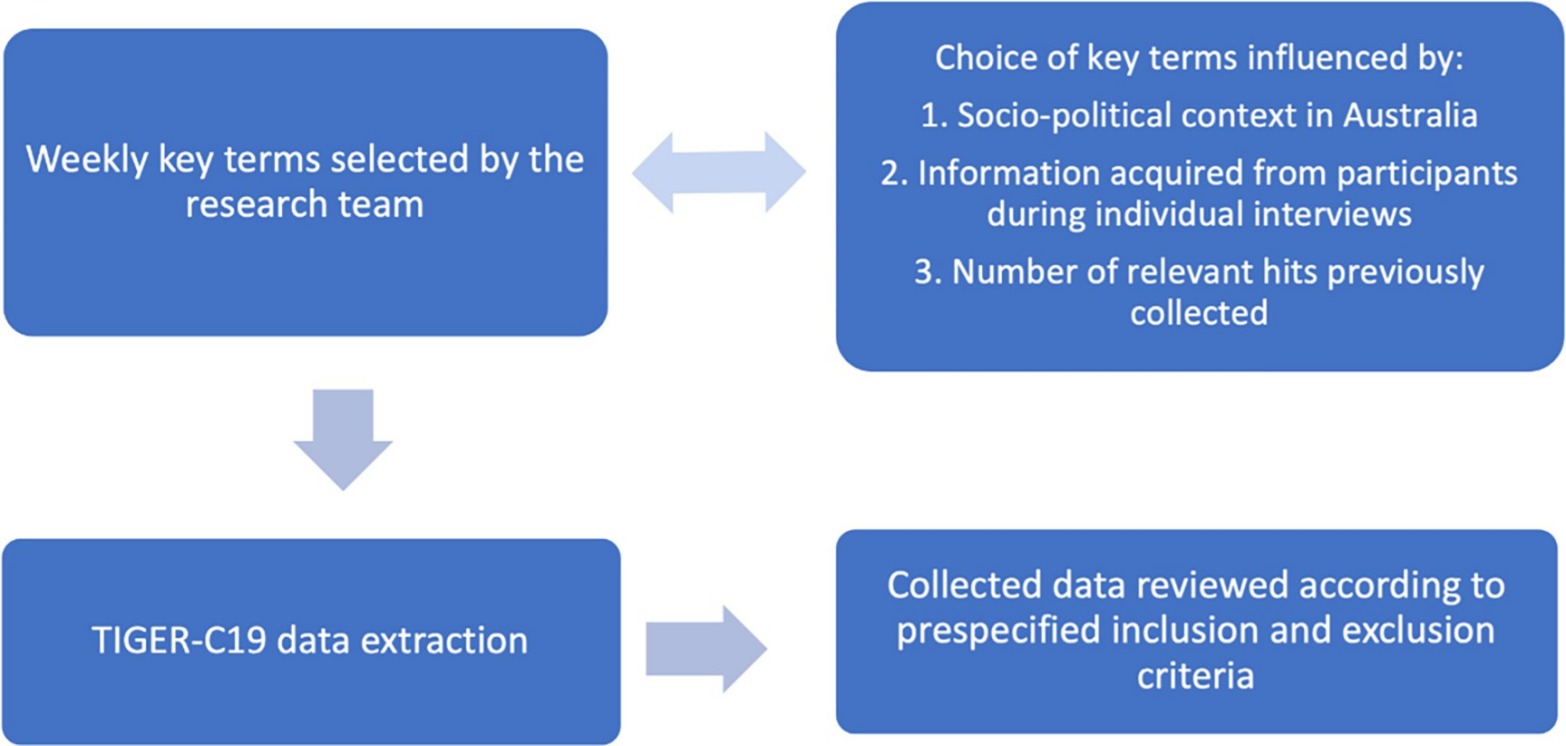
Social media key term selection process.

Weekly data collection occurred using the TIGER-C19 tool over eight weeks from June to July 2021. Each week selected search terms aimed to extract posts that gave insights into the community attitudes and understanding of COVID-19 in pregnancy in Australia. Search terms evolved and changed as results were reviewed each week. Search terms that did not yield large numbers of results were modified and new key terms identified, these changes were also made in response to the evolving COVID-19 situation in Australia and changing socio-political context. For example, when policy changes recommending vaccination for pregnant women came into effect, key terms were adjusted to include vaccines and vaccination, see Fig 2. Key search terms used included: pregnancy, pregnant, COVID baby, miscarriage, infertility, vaccine, vaccination, antenatal, birth and breastfeeding. To ensure results were relevant to the research topic, these key terms were often searched for in conjunction with COVID specific terms, such as ‘vaccine + miscarriage’, or ‘pregnancy + vaccine’.

#### Data analysis

Inductive content analysis was used to interpret the social media data [28]. Inductive content analysis allowed for social media data to be considered broadly and for conceptualisations to be made without pre-set categories. Data were analysed for common patterns as they relate to pregnancy and COVID-19. The 220 most re-tweeted (Twitter), and up-voted (Reddit) posts that met the pre-specified inclusion criteria were included in the analysis. These posts were initially read and assigned a code using an Excel spreadsheet and Word document. One post could be assigned to multiple codes. Codes were then grouped into broader categories which became the coding framework for subsequent coding. This was an iterative process, with codes and categories being reviewed and refined as needed. Analysis occurred with the wider research team through regular meetings to discuss coding frameworks and resolve discrepancies in interpretation.

### Interviews

#### Sample and recruitment

Individual interviews with pregnant and postnatal women were conducted with 21 women. Interview participants were recruited across Australia through online social media advertising posts. Potential interviewees contacted the primary researcher (CC) by email and were provided with a Plain Language Statement. They were then invited for an interview if they met the inclusion criteria: pregnant since March 2020; Australian resident; aged at least 18 years; proficient in English; and with access to a computer or phone for interview.

#### Data collection

Individual semi-structured interviews were conducted by an experienced qualitative researcher using a semi-structured interview guide via the video platform ‘Zoom’ or by telephone, as preferred by participants. Lasting between 30–60 minutes, interviews were audio recorded and transcribed verbatim. Verbal consent was obtained and demographic information collected. The primary researcher (CC) conducted all interviews. Interviews discussed the information participants had received regarding impacts of COVID-19 on pregnancy, where information was sought, the use of social media in obtaining and sharing information and how information received shaped experiences and decision-making. The semi-structured nature of interviews allowed participants to place emphasis on experiences most important to them. This provided important insights into current public perceptions about pregnancy and COVID-19 and informed search terms used in the social media analysis. We stopped recruitment after we achieved information power [29], minimal new data were being generated and we had sufficient data to answer the research question.

#### Data analysis

Thematic analysis was used to analyse interview data [30]. Thematic analysis is suited to analysing transcribed interviews, where detailed and lengthy data can be coded to generate patterned meanings [30]. Coding and interview data were managed using NVIVO software [31]. Analysis began by reading through all transcripts to become familiar with the data set. Two researchers (CC, AW) individually coded a sub-sample of transcripts and codes were contrasted and compared for consistency. Coding discrepancies were resolved through discussion and collaboratively reviewing the data with the wider research team. The remaining transcripts were coded by the primary researcher (CC). This was an iterative process with codes being refined and developed. Codes were then grouped to create initial themes and sub-themes and the data set was re-read to ensure the data depth was reflected. The analysis process occurred in conjunction with the wider research team through weekly meetings and discussions, improving the richness of interpretations.

## Results

### Social media

Over an eight-week period, between June and July 2021, 17, 689 posts were extracted from Twitter and 2,523 posts from Reddit using TIGER-C19. The top 220 most re-tweeted and up-voted posts that met inclusion criteria were included in the final analysis. Through content analysis seven main categories were identified. Results and selected quotes are presented in Table 1 accompanied by the social media platform used. Quotes have been paraphrased to avoid identification of social media users. Results provide insight into what information was being shared, sought, and discussed on social media about pregnancy and COVID-19 during the pandemic in Australia.

**Table 1:** Results of social media analysis.

Category	Description	Examples
Sharing and seeking information	Most posts in this category shared important updates and information with other social media users. This included updated recommendations for COVID-19 vaccination in pregnancy. Users shared and sought information regarding the definition of a ‘carer’ during lockdown, eligibility for vaccination in different states and pregnancy and COVID-19 related resources.	*The college of general practitioners now recommends the vaccine at all stages of pregnancy- Reddit user* *Great*, *I checked a few weeks ago and the advice was to not get vaccinated*, *thanks so much for letting me know the [RANZCOG ATAGI] update*! *I’ll definitely be speaking to my obstetrician to see if I can get it [the vaccine] before the baby arrives*.*–Reddit user* *I’m from WA (the state of Western Australia) and we haven’t had much exposure to COVID at all*, *so I don’t know much about the vaccines*, *this might sound random*, *but can you get your second vaccine while pregnant*?*–Twitter user*
Confusing and/or insufficient information	Many posts highlighted confusion about the safety and eligibility for vaccination in pregnancy as well as conflicting messages from individual practitioners and health and government organisations.	*I remember when the chief medical officer was recommending against vaccination during pregnancy*. *I think there needs to be a better explanation as to why that advice has changed beyond their statement–Reddit user* *Pregnant women need clearer messaging [about the vaccines]*, *COVID-19 makes risk assessment complicated for women who want to be or are pregnant–Reddit user* *Pregnant women are being mum-shamed on social media by anti-vaxxers*, *saying things like “you’re crazy” or “protect your baby” while our government is silent*, *providing no clear guidance to support pregnant women or health workers–Twitter user*
Impacts on maternity services	Many posts highlighted disruptions to maternity care during the pandemic. These included visitor restrictions in hospital and difficulty accessing antenatal and postnatal services. Some posts also highlighted certain benefits to these disruptions.	*I just gave birth to my first child; I wish I could have some visitors*!–Reddit user *Be glad you don’t have to go to an in-person appointment the parking at the hospital is terrible- Reddit user* *I’m 38 weeks and have been so ready and excited to give birth*, *but I can’t help but start to feel anxious… I just want to know my partner will be able to stay with us if the baby is born in the next week*, *I’m desperately hoping this lockdown works and won’t be extended–Reddit user* *I want to have a vbac [vaginal birth after caesarean section] but this is making me want to get a c-section [caesarean section] at a hospital I know so I don’t have to go to a hospital I’ve never been to before*, *meeting all new people and hoping they will do the right thing by me*: */–Reddit user* *I’m so anxious that I’m not getting doppler checks or being seen face-to-face by my health provider*. *I will have no idea if this baby is even alive until I go for my scan–Reddit user*
Promoting vaccination in pregnancy or breastfeeding	Posts frequently highlighted the safety and benefits of getting vaccinated during pregnancy or breastfeeding. This comprised of both personal accounts of being pregnant and getting vaccinated and advocacy efforts for pregnant people to be considered a priority population for vaccination.	*I really hope that the eligibility of those pregnant or breastfeeding in Australia to get Pfizer is reviewed in alignment with the statement from ATAGI and RANZCOG–Twitter user* *I am 20 weeks pregnant*, *and after getting some advice from my health provider*, *I had my COVID-19 vaccine yesterday*.–*Twitter user*
Risk/benefit and decision making	Posts in this category highlight both perceived benefits and risks for pregnant people considering vaccination. Many were motivated to get vaccinated due to the possibility of passing on immunity to their newborn. Posts in this category also included discussion about pregnancy intentions alongside the pandemic.	*Research has now shown pregnant women can pass antibodies onto their babies and that the vaccine does not cross the placenta*. *I would choose to get the vaccine because of the risk of complications from COVID*.*–Reddit user* *If the Australian government is sending conflicting messages [about the vaccine] why risk it*? *The chances of you catching COVID in QLD [the state of Queensland] while we’re locked down is tiny… Very few women vaccinated in the first trimester have carried their babies to term so there’s not much data out there about how safe it is*. *It’s a small risk but the chances of you getting COVID are even smaller*.*–Reddit user* *I’ve seen loads of American women in my pregnancy forums saying they’ve had the vaccine while pregnant with no issues and their babies are born with antibodies*, *which I think is fantastic*! *I plan to get vaccinated if I can while I’m still pregnant–Reddit user*
Misinformation	There was evidence of misinformation circulating online. These posts predominately related to the safety of the COVID-19 vaccines in pregnancy.	*Before you think of getting vaccinated*, *know that 60% of Americans are refusing this experimental vaccine*. *In Australia one person has died from COVID*, *a few hundred from the vaccine and over 20*,*000 adverse reactions recorded*. *In London pregnant women are having stillborns*, *understand*?*–Twitter user* *A heavily pregnant woman was just on live television saying how she’s just had the COVID-19 vaccine and that she’s planning the have the second dose at 38 weeks*! *And she’s promoting this to other pregnant women and saying it’s safe*, *this is very evil propaganda–Twitter user* *How can these untested drugs [COVID-19 vaccine] be pushed onto pregnant women*, *it’s revolting–Twitter user*
Difficulty acquiring vaccination	Many women expressed difficulty accessing vaccination when desired. Barriers to vaccination included long wait lists and ineligibility, this caused frustration and distress.	*I hope there aren’t any further outbreaks because I can’t get vaccinated due to my age and I’m pregnant*, *I have to wait until later in the year when maybe my doctor will let me get it–Reddit user*

### Interviews

A total of 21 interviews with pregnant and postnatal women were conducted from June to July 2021. Participant demographics are described in Table 2; all participants identified as women. Through thematic analysis four key themes were identified: dealing with the emotional impacts; searching for knowledge; turning to informal sources; and communicating effectively. Verbatim quotes are accompanied by the stage of pregnancy and number of children. Information in brackets has been added by the researcher for additional context.

**Table 2:** Demographic characteristics of interviewed participants *(N = 21)*.

Characteristics	Participants (n)
Age in years, mean (range)	32 (25–43)
Country of birth	
Australia	15
Overseas	6
State/Territory of Residence	
Victoria	7
New South Wales	5
Queensland	3
South Australia/Western Australia/Tasmania/Northern Territory/ACT	1/2/1/1/1
Education	
High school	1
Bachelors/Diploma	16
Masters/PhD	4
Regional/Inner city	
Metropolitan	19
Regional/rural	2
Stage of pregnancy	
Pregnant at time of interview	5
Postnatal at time of interview	16

#### Dealing with the emotional impacts

Most women expressed difficulty finding information about the impact of COVID-19 on their pregnancy, birth, and after birth. They felt anxious, frustrated, and concerned when they were not provided with information that was important to them. One woman described how limited access to COVID-19 information impacted her pregnancy *“It made it a little bit more stressful than it needed to be*. *Not knowing*, *sort of feeling like you’re in the dark a little bit and there’s no one who’s in your corner*.*” (Postnatal mother*, *first baby)*. For some, this lack of information directly contributed to negative experiences during pregnancy and birth; *“it had an impact to the birthing experience*, *because it [lack of information] made probably an experience [birth] more negative*… *because I had no idea what to expect*, *with COVID regulations*.*” (Postnatal mother*, *second baby)*

In contrast, when women were provided with information about COVID-19 they reported both satisfaction and a sense of reassurance. Information was generally provided through their primary maternity provider—General Practitioner (GP), midwife, or obstetrician. Some described how this information influenced their decision to adopt personal precautions and where to accept health care. *“I think all my information about COVID*, *and pregnancy probably came through her [my obstetrician] …she talked a lot about how I could manage being pregnant in this situation and keeping safe*.*” (Postnatal mother*, *second baby*.*)* Providing COVID-19 information to women empowered them with an increased sense of control and the ability to adopt risk reduction strategies.

#### Searching for knowledge

*Risks to themselves*, *their pregnancy and the baby*. Participants actively sought information regarding the potential impacts and associated risks of COVID-19 on their pregnancy and/or newborn. Many participants wanted to know common signs and symptoms of COVID-19 infection in pregnancy and/or newborns and described difficulty finding this information: *“There’s a lot of fear*. *What happens if I do get COVID*? *Is that gonna affect my pregnancy*? *… how’s that gonna affect the baby potentially*?*” (Postnatal mother*, *second baby)*. Rapid changes to maternity care impacted on women’s ability to access routine antenatal information: *“So there was no access to birthing classes*, *any of that sort of stuff…we were just winging it basically*.*” (Postnatal mother*, *first baby)*. This extended into the postnatal period with many women reporting not receiving child maternal health visits during periods of lockdown and having difficulty sourcing important information about newborn care. *“I didn’t have the normal maternal and child health visits*, *how do you care for a newborn… what do you do when your child has a fever and you’re in lockdown… I just wanted her [child] to see a GP but she couldn’t until you’ve had a COVID test*… *and back then they were taking four to five days*, *what’s happened to your freaking baby in the meantime*?*”*. (*Postnatal mother*, *second baby*).

Participants also described receiving limited information about the COVID-19 vaccine and cited this as a major gap in information provided to them. Many were uncertain whether it was safe or recommended in pregnancy. *“There hasn’t actually been any proactive messaging to me about COVID-19*, *especially regarding the vaccines there’s been nothing mentioned to me or discussed with me or with any of the health providers*, *if that would affect me*, *or even if I would be able to get the vaccine*.*” (Pregnant woman*, *first pregnancy)*. Public health recommendations and advice changed over the course of the interview period and often these updates were slow to reach women in the community, with many participants being unaware of changes to vaccine recommendations.

*Practical implications*. There was a need for better communication on the impacts of changes to maternity care. This was a consistent theme from women in settings of both higher and lower levels of COVID-19 community transmission. Restrictions often meant limited contact with health services where women would give birth and this presented a unique gap in women’s knowledge of the hospital they would receive care in. Women wanted practical information, for example, tours of maternity and birthing suites, videos and maps displaying the best place to park the car and enter the building and how to get to the maternity ward:*“I was even hoping if just a midwife with a phone went around and did like a video*, *labour room*, *birthing area*, *like [a] tour just to give us an idea*, *because I literally have no idea how to even get into the hospital to find the birthing ward*, *what did the rooms look like*? *…we had nothing*, *it was very daunting*.*” (Postnatal mother*, *first baby)*.

Many women were confused or unaware of the impact of hospital restrictions on labour and birth. They received inconsistent information from staff members and reported confusion as to what constituted a ‘support person’ or ‘carer’. Restrictions regarding support people were particularly unclear for non-heteronormative family units: *“No that [hospital restrictions] was communicated terribly… or it just wasn’t communicated at all*. *before I went in to have the baby I asked about the restrictions*, *and the nurse on maternity just didn’t even know… because I didn’t have a husband coming in you know*, *so what does [my] mum count as and can she bring back my son and they just were clueless*.*” (Postnatal mother*, *birth to twins)* Clarity of this information was especially important for participants with complex pregnancies or with previous experiences of miscarriage who spoke of the importance of knowing their support person would be allowed to attend appointments. The impact of being COVID-19 positive on the level of care birthing women required or being separated from their newborn was also of concern, this was especially relevant for those from regional locations or smaller states where local hospitals may not be able to provide appropriate care to a pregnant woman who was COVID-19 positive. *“I googled the s—t out of*, *you know*, *what would happen if I got COVID-19 while I was pregnant*. *What would happen if I ended up having it [COVID-19] while I was going in to give birth … would they take her away if I was diagnosed positive*.*” (Postnatal woman*, *third baby)*.

#### Turning to informal sources

When desired information was insufficient, inaccessible, or unavailable, women turned to informal sources such as internet searches, social media, friends and family. Although many wanted information from reputable sources, barriers limited their ability to attain this information. This included difficulty navigating government websites and fear information available would be outdated: *“I would pass New South Wales Health [department]…I don’t think they’ve been very good actually at presenting like digestible information” (Pregnant woman*, *second pregnancy)*.

All participants used some form of social media. The most cited platforms were Facebook and Instagram, although participants also referred to Reddit, TikTok, Snapchat and Twitter. Women described using social media as an additional source of information when information was otherwise limited: *“the information in the public domain wasn’t really that much…I guess that’s what Facebook kind of gave me is that broader social network to ask questions or to at least have information that wasn’t in the general publications*.*” (Postnatal mother*, *second baby)*. Social media was a useful resource for missed health information. These women followed health providers and maternity influencers on platforms such as Instagram. For example, one woman said: *“What I did seek from missing out on antenatal classes lactation consultants and midwives that are now providing their services through Instagram*, *and they will post*, *ask me anything type of stories*.*” (Postnatal mother*, *first baby)*. Social media algorithms also had the potential to present information to users as well as suggesting other pregnancy and birth pages to follow. In this way, social media users were passively exposed to new information. *“Probably not sourcing it out*, *it would just pop up on Facebook*, *because I’ve obviously googled something*… *So then I would just click on something from there”*. *(Pregnant woman*, *second pregnancy)*.

Social media groups such as those found on Facebook were widely used and many described them more accurate and timely than information sought through official channels. This was especially true when information related to the impact of restrictions on health service provision: *“sometimes because you have to call the antenatal place and you’re usually on hold for like an hour*, *so we just check there [Facebook group] first because people answer faster*.*” (Postnatal mother*, *second baby)* Many noted first seeing important updates, including recommendations on COVID-19 vaccinations during pregnancy, on their social media groups. These interactions also functioned as a source of support while navigating the COVID-19 pandemic. *“There was actually three of us that were pregnant all at the same time …if they found anything helpful*, *they’d send me a screenshot*, *or we often talked about like*, *it’s going to be okay kind of thing…It was hard for any family to be around us*. *It was hard for them to understand*, *they weren’t pregnant in a pandemic*.*” (Postnatal mother*, *first baby)*.

Participants expressed concern about misinformation present within social media groups. Misinformation was often related to the safety and potential side effects of COVID-19 vaccines: *“people saying that it [vaccine] increased the risk of miscarriage and stuff*. *I mean*, *just the sorts of things you’d kind of expect to be said in terms of misinformation*.*” (Pregnant woman*, *first pregnancy)*.

#### Communicating effectively

Participants identified several ways in which communication could be improved. This included integrating COVID-19 information into routine antenatal and postnatal care, preparing information in advance for lockdown periods and creating more opportunities for online interactions with health providers.

*Normalising and preparedness*. Health providers were trusted sources of information. Many felt that just as other common illnesses such as influenza are discussed routinely in antenatal appointments, COVID-19 information and vaccination should be proactively shared and integrated into routine antenatal care: *“It’s about normality at this stage*, *whether the messaging should just address it as any other illness that could potentially impact pregnancy*, *like*, *for example*, *flu*, *you got to get your flu shots through pregnancy…That sort of messaging*. *So it’s probably about normalising*.*” (Postnatal mother*, *first baby)*. The hospital website was seen as an extension of trusted health providers and most participants highlighted that websites should be a primary source of readily available information but were often disappointed with the level of information provided.

Women also expected health services to prepare for the event of snap lockdowns and rapid changes to the provision of maternity care and have information ready to disseminate. *“It will be good for them to have things ready to hand out and send out*… *Because we can see how quick the snap lockdowns happen*… *they need to have information ready to give out to the people who are going to be giving birth*.*” (Postnatal mother*, *second baby)*. This information should be provided through multiple communication methods both passively and actively through social media posts, updated websites and personal communication to women through an email or text.

*Interactive and in real-time*. A consistent theme throughout interviews was the importance of making information more interactive when face to face care was restricted, such as during lockdowns. Participants often valued zoom sessions over information sheets and video appointments were preferred over telephone or telehealth appointments. *“So I really wanted to do in person antenatal classes*, *to ask the questions*, *etc*. *And yeah…just make it like zoom meetings*, *the PowerPoint presentations just make it more impersonal*. *Plus*, *you just can’t ask the questions that come up with as you go*.*” (Postnatal mother*, *second baby)*.

Regular live sessions where pregnant women could ‘ask anything’ and ‘real time’ interactions with midwives were suggested as important ways to increase interaction between health services and providers. Participants highlighted social media pages with live streams as one preferred platform and gave examples of when this had been done well. *“I follow the hospital on Instagram*. *And they’re pretty good*, *it’s sort of communicating changes very*, *in a very timely manner*. *So they will*, *anytime the rules changed*, *you know*, *within a couple of hours*, *they would have posted on Instagram saying*, *here’s the new rule*. *And I think that was quite good*.*”* (*Postnatal mother*, *second baby*) Participants described following trusted sources on social media and wanting regular communication, even if no new updates were warranted. *“I just feel like*, *tell us what’s going on*, *let us know that you’re dealing with it*, *or let us know what is that it’s being investigated or whatever*, *but don’t pretend it’s not happening*.*” (Postnatal mother*, *second baby)* If there was uncertainty, women wanted this acknowledged and some indication that this information would be made available once decisions had been made.

## Discussion

Findings from this study highlight the difficulty pregnant and postnatal women had in accessing timely and clear information during the COVID-19 pandemic in Australia. Many women found the lack of information available to them to be frustrating and a source of increased anxiety, this was compounded by health related anxiety already caused by the pandemic, and this is reflected in other studies [32–34]. However, when information was provided it empowered women to adopt risk reduction strategies and make informed decisions about their pregnancy and birth. Social media provides an ideal platform for the rapid dissemination of targeted information and most women followed at least one trusted source on social media. However, the use of social media also has the potential to increase exposure to misinformation which can impede the management of disease outbreaks [35]. To combat this, our findings demonstrate the need for ongoing timely, interactive, and proactive health communication from government organisations and health services.

Participants perceived that information on COVID-19 vaccines, the impact of restrictions on service provision and the effects of COVID-19 on pregnancy and newborns was limited and difficult to find. Indeed, difficulty finding information regarding the impacts of COVID-19 on pregnancy have been documented elsewhere [34]. A recent study of pregnant women in Sweden showed many felt that they did not receive sufficient information from health care providers during the pandemic which increased feelings of stress and uncertainty [36]. In addition, an Australian cross sectional study found that only one-third of pregnant women surveyed received antenatal education, and missing this information was seen as negatively impacting on birthing experiences [33, 37]. These information gaps are significant, as information provided during maternity care has been shown to be an important factor in determining maternal satisfaction [38, 39], and insufficient COVID-19 information in this study was shown to directly contribute to negative experiences during pregnancy and birth. Even when information was available, many women found it confusing. This may in part be due to the evolving nature of COVID-19 situations however, two recent studies found that most COVID-19 information provided to the Australian public exceeded recommended reading levels required to ensure widespread understanding [40, 41]. This is reflected in findings from the social media analysis which demonstrated a strong need for clearer information, especially with regards to vaccination.

This study also highlights the importance of familiarity with place of birth for pregnant women, people and support persons. Many women were disconnected from health services in which they would labour and birth due to public health restrictions. Women described wanting to better ‘know’ the hospital in which they would give birth, for example where to park, how to get to the maternity department and being able to see the labour ward. Not ‘knowing’ this practical information exacerbated feelings of uncertainty and a lack of control already amplified by the COVID-19 pandemic. Many of our participants were also unable to access antenatal education due to the pandemic. This is important, as having a sense of control during labour and birth, which is also facilitated by the provision of antenatal education, has been linked to increased satisfaction and a more positive birth experience [42, 43]. Although previous studies have examined the concept of a women’s sense of control in pregnancy and birth, this study highlights the role familiarisation with the hospital environment has on the birthing experience, and is consistent with previous Australian studies [44, 45]. Our findings clearly suggest that understanding the schematics of the hospital setting during times of disconnect could improve women’s sense of control and preparedness for birth. Where external circumstances impede on women’s ability to visualise and become familiar with place of labour and birth, health services should attempt to fill this gap by finding alternate ways to support birth preparation through provision of online resources.

Social media has been shown to be an important source of information for pregnant women during the pandemic and most participants in our study described frequently using social media and considered it to be a preferred source of information [32, 34, 37]. Despite this, many health services and providers under-utilised social media to communicate information and changes. This is despite the high acceptability of social media based antenatal care services, and other studies that highlight the acceptability and preference of pregnant women to receive information in online forums [37, 46]. During periods of increased COVID-19 transmission, we found participants favoured health services that provided prompt social media updates regarding their response to changing circumstances and were critical of the lack of communication from many government bodies, health organisations and services. On the 9^th^ June 2021 The Royal Australian and New Zealand College of Obstetricians and Gynaecologists (RANZCOG) and the Australian Technical Advisory Group on Immunisation (ATAGI) released a joint statement recommending vaccination in all stages of pregnancy for the first time in the Australian setting [47]. This statement was released during data collection and many participants interviewed after this release were unaware of these changes. This demonstrates the current limitations peak health and government bodies have in reaching the Australian community and the need to further develop communication strategies. Given the potential negative impacts of COVID-19 on newborns and those who are pregnant and the rising maternal mortality rates seen globally as a result of the pandemic [7, 48, 49] it is integral that organisations and providers facilitate the dissemination of important updates through platforms like social media, to ensure information reaches the target population.

### Implications for policy and practice

Online resources should be prepared and ready for use in the event of future public health measures related to COVID-19 and other potential public health crises. Health services and providers should prioritise the use of alternative, interactive information sessions–for example, virtual (Zoom-based) antenatal classes and regular online ‘ask me anything’ sessions—rather than pamphlets and information sheets. Where a physical disconnect between health services and pregnant women exists, practical resources about where to park and how to access maternity wards should be provided to better support women when they access unfamiliar services during labour and birth. Health providers are trusted sources of information [50] and information regarding COVID-19 and the vaccine should be routinely integrated into antenatal care. Providers should be aware that online social media groups are common sources of pregnancy information for many people and may consider participating in large social media pregnancy groups within their local areas to address misinformation and to engage with pregnant women and people in their communities.

It would be helpful for health services to establish and make readily available protocols outlining how varying public health measures will impact on maternity care. The presence of support persons during antenatal appointments and birth is highly valued by pregnant people [37] and protocols should include clear definitions of what constitutes a ‘support person’ and should consider family units that do not adhere to heteronormative relationships. When circumstances rapidly change, proactive statements form health services acknowledging change and their strategy to address this change could allay anxiety and uncertainty experienced by pregnant women and people during these situations [51].

### Strengths and limitations

The TIGER-C19 data analytics tool only extracts data from social media platforms Twitter and Reddit and is not representative of all social media users. The top upvoted (Reddit) or re-tweeted (Twitter) public posts were analysed, which may have skewed selected posts to those with larger public profiles. Unlike Twitter, Reddit users are largely anonymous so it is difficult to ascertain the origin of the user and international users may have been unintentionally included. Judgements about the meaning of social media posts were made by the research team and there may be some misinterpretation. The potential for misinterpretation was minimised through discussions among the research team. We did not track the author of each post, and it is possible that users who were vocal may have been included multiple times. Although the novelty of the methods used may be a limitation, it can also be viewed as a strength where the use of concurrent social media analysis and in-depth interviews acted to validate and increase the credibility of our findings. Another strength of this study included representation of women in interviews from all Australian states and territories, which provides some insights to experiences nationwide.

## Conclusion

The provision of comprehensive, accurate and timely information is integral to support pregnant and postpartum women during the COVID-19 pandemic. A lack of information increased feelings of distress and had the potential to negatively influence experiences of pregnancy and birth. Social media was viewed as a highly acceptable method of communication and provides an opportunity for health services and providers to engage and interact with women in the community and communicate rapidly changing information in real-time.
